# A Systematic Review of International Bereavement Models of Care and Implementation Barriers and Facilitators

**DOI:** 10.1177/00302228251315502

**Published:** 2025-01-21

**Authors:** Nicci Bartley, Luna Rodriguez Grieve, Claire Cooper, Laura Kirsten, Cindy Wilson, Betsy Sajish, Joanne Shaw

**Affiliations:** 1 Psycho-oncology Co-operative Research Group, School of Psychology, 4334 The University of Sydney, Camperdown, NSW, Australia; 2 Nepean Cancer and Wellness Centre, Nepean Hospital, Kingswood, NSW, Australia

**Keywords:** bereavement, grief, model of care, implementation, systematic review, grief

## Abstract

Bereavement care can facilitate adjustment to death and reduce immediate distress and long-term morbidity, mortality and health service utilisation. This systematic review aimed to identify international models of bereavement care, and barriers and facilitators to implementing such models. A systematic search of MEDLINE, Embase, CINAHL and PsycINFO identified 64 studies for inclusion. The most common forms of bereavement support were bereavement packs, memory-making activities, condolence cards, memorial services, and follow-up contact. Only 14 models included a formal assessment of complex grief, and 17 studies considered culturally and linguistically diverse populations. Barriers included lack of institutional/financial support, staff discomfort delivering care, lack of dedicated staffing, and difficulty collecting/maintaining information. Facilitators were adequate funding/infrastructure, formal protocols/procedures, dedicated staffing, and staff training and support. Future research should address bereavement models of care in settings beyond palliative care, considering culturally and linguistically diverse populations, and should provide implementation data and strategies.

## Introduction

Bereavement is a multifaceted natural response to death and dying that includes emotional, cognitive, physical, spiritual, and behavioral reactions (Bui, 2018; Morris & Block, 2015). Approximately 9.8% of bereaved individuals will not adapt to their bereavement and will experience complicated grief or Prolonged Grief Disorder (PGD) (Lundorff et al., 2017). Risk factors for PGD and poor psychological wellbeing include background factors (e.g., closeness to the dying person, previous mental health issues, or minority ethnic status), treatment-related factors (e.g., aggressive medical intervention, family conflict regarding treatment, or caregiver burden) and death-related factors (violent death, multiple losses in quick succession, or dissatisfaction with death notification) (Hudson et al., 2018; Lobb et al., 2010).

Bereavement care can facilitate adjustment to death (Agency for Clinical Innovation, 2021; Grant et al., 2021) and can reduce immediate distress and long term morbidity, mortality, and health service usage (Grant et al., 2021; Morris & Block, 2015; Penny, 2017). Types of bereavement support include: information about bereavement and relevant supports provided by family, friends, and health professionals; non-specialist support from trained volunteers, peer support, or community organizations; and professional specialist support provided by mental health services, bereavement services or psychologists (Aoun et al., 2012). Ideally, the level of bereavement support is delivered based on need. For those who have a low level of support needs, such as individuals experiencing normal grief, provision of information about bereavement and relevant supports are recommended. Whereas, more intensive support needs, such as specialist supports can be reserved for those with more complex needs (Aoun et al., 2012; Lobb et al., 2010).

In Australia in 2022, there were more than 170,000 deaths (Australian Bureau of Statistics, 2023). The majority of deaths in Australia occur in medical settings, making bereavement support in the health service setting integral (Australian Bureau of Statistics, 2021). However, evidence-based bereavement care is not routinely provided or delivered by trained staff (Breen et al., 2014; Grant et al., 2021). Models of care are comprehensive approaches that can guide the organisation and delivery of care in a health service (Brereton et al., 2017; Luckett et al., 2014). Models of bereavement care outline best-practice bereavement care, the different level of care provided routinely within a health service and describes when, how and by who, care is delivered. Implementing a bereavement model of care in a health service should result in routine provision of evidence-based bereavement care by appropriately trained staff.

Bereavement literature focusses on delivering specific bereavement interventions to specific populations, for example memory making activities for pediatric deaths (Cole et al., 2020). There is limited literature focused on bereavement models of care or programs across health services, or the implementation of such models of care. Further much of the literature is medicalized with little focus on psychosocial models of bereavement care that are applicable to diverse communities (Grant et al., 2021).

The aim of this systematic review was to identify international health service models of bereavement care, and barriers and facilitators to implementing such models of care.

## Methods

### Study Design

This systematic review protocol was designed and conducted using the Preferred Reporting Items for Systematic Reviews (PRISMA) standards. The review was registered with PROSPERO, registration ID CRD42023401125.

### Search Strategy

A systematic search of MEDLINE, Embase, CINAHL and PsycINFO was conducted in March 2022. Search terms were a combination of keywords and MeSH terms relating to bereavement, care delivery and health services (Table 1). An updated search was conducted in April 2024. References were also screened, and relevant studies included.

**Table 1.** The Systematic Review Search Strategy.

	Keywords and MeSH terms
Bereavement	Bereave*, Grief*, Prolonged grief disorder, Griev*, Mourn*
Care delivery	Support program*, Support service*, Psychosocial intervention*, Psychological intervention*, Hospice care*, Hospice program*, Palliative care, Bereavement care, Bereavement support, Model*, Service*, Care, Follow-up, End-of-Life, Risk assessment, Best practice*, Guideline*, Principle*, Stepped care, Tier*, Evidence-based-practice, Evidence based practice
System	Health service*, Health care service*, Healthcare service*, Hospital*, Institution, Hospice*

The inclusion criteria for the review were English-language publications of original research that presented bereavement models of care or programs for adults grieving the loss of a loved one in a health service setting. Studies that: were not in English, did not include original research (literature reviews, commentaries, letters to editor, conference abstracts, conference proceedings), applied a single intervention, assessed bereavement needs/quality of death, were focussed on other populations (e.g., healthcare professionals, children/adolescents) or settings (community based), or focussed on losses other than the death of a human loved one (e.g., death of pets, divorce) were excluded.

The search strategy identified 9554 studies. After removing duplicates, the remaining 6318 articles were screened by title and abstract (JS, NB, CC), each study was double screened. The three authors had an overall agreement rate of 79% (Cohen’s kappa 0.44). The full texts of 429 retained papers were retrieved for double screening by JS, NB, CC and LRG, resulting in the exclusion of 372 articles that did not meet the eligibility criteria: publication type (e.g., conference abstracts); wrong outcomes (e.g., bereavement care needs); wrong intervention (e.g., bereavement intervention rather than model or program of bereavement care); wrong study design (e.g., inadequate description of the program/model of care); wrong setting (e.g., bereavement care in community-based settings); wrong population (e.g., focus on supporting healthcare professionals). Discrepancies were resolved by consensus. Where the full text of an article was unavailable through the authors institution, article authors were contacted for full texts via email or ResearchGate. Only three articles were retrieved via this method. Of these, 57 met eligibility criteria and were included for data extraction and quality assessment. An additional seven studies were identified through reference screening (Figure 1), resulting in 64 studies included for data extraction.

**Figure 1.**
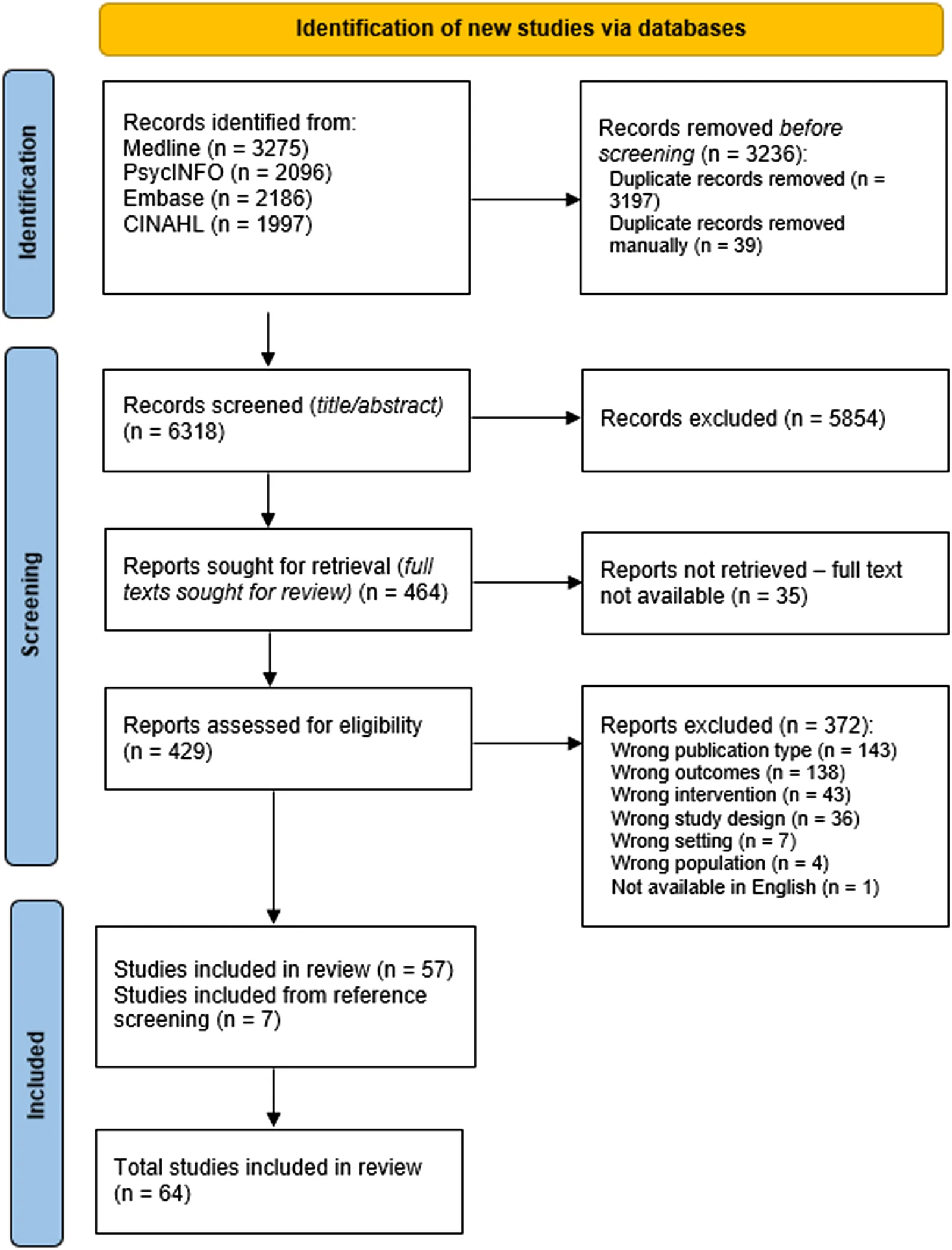
PRISMA diagram for systematic review of literature.

### Data Extraction and Assessment of Study Quality

For each publication, data was extracted following the Preferred Reporting Items for Systematic Reviews (PRISMA) standards. Data extracted included study design, objectives, population, and bereavement model of care features including services provided, staff involvement, support and training, screening and risk assessment, cultural considerations, evaluation of services, and barriers and facilitators. Data was extracted independently by two authors (LRG and NB) with an overlap of 38% to ensure reliability. All studies included were evaluated using the Mixed Methods Appraisal Tool (MMAT). One author (LRG) independently assessed study quality with a 47% overlap assessed for quality by a second author (NB). There was a high level of agreement assessing study quality (Cohen’s kappa 0.87). All disparities were resolved through discussion.

## Results

### Study Characteristics

Of the 64 studies included, 22 were descriptive, outlining an existing bereavement model of care, 21 were mixed methods, 12 were qualitative, and nine were quantitative. Most of the studies included were conducted in the United States, but five published Australian research. Most studies were based in an ICU/palliative care/oncology setting, followed by paediatrics, peri/neonatal, hospital wide, emergency/acute, and less commonly psychiatric, and surgery settings. Thirty-one studies included bereavement from both sudden and expected deaths, 23 studies focussed on expected deaths, and ten studies on sudden deaths. The bereaved population included in the studies was predominately any bereaved loved one. The study characteristics for each study are provided in Table 2.

**Table 2.** Study Characteristics of Literature That met the Systematic Review Inclusion Criteria.

Study	Country	Study type	Health setting	Type of death	Bereaved population
Adamowski et al. (1993)	Canada	Quantitative	Emergency/acute	Sudden	Anyone
Aho, Astedt-Kurki, Tarkka, & Kaunonen, (2011)	Finland	Mixed Methods	Paediatric	Both	Parents
			Hospital-wide		
Aho, Tarkka, Åstedt-Kurki, & Kaunonen (2011)	Finland	Mixed methods	Peri/neonatal	Both	Parents
Berrett-Abebe et al. (2017)	United States	Qualitative	ICU/palliative/oncology	Expected	Parents
			Paediatric		
Blackburn and Dwyer (2017)	Australia	Descriptive	ICU/palliative/oncology	Expected	Anyone
Bucaro et al. (2005)	United States	Descriptive	Paediatric	Sudden	Parents
Buchanan et al. (1996)	United States	Qualitative	Trauma	Sudden	Anyone
Burke and Gerraughty (1994)	United States	Mixed Methods	ICU/palliative/oncology	Expected	Anyone
Chaplin (2003)	United Kingdom	Qualitative	Hospital-wide	Both	Anyone
Cole et al. (2020)	United States	Descriptive	Peri/neonatal	Expected	Parents
Coolican and Pearce (1995)	United States	Mixed Methods	Emergency/acute	Sudden	Anyone
Cooper et al. (2020)	United States	Qualitative	Emergency/acute	Sudden	Anyone
deJong-Berg and deVlaming (2005)	Canada	Descriptive	Paediatric	Expected	Parents
Edi-Osagie and Evans (2005)	United Kingdom	Descriptive	ICU/palliative/oncology	Expected	Parents
			Peri/neonatal		
Edwards (2020)	United Kingdom	Descriptive	Paediatric	Both	Anyone
Farrow and Felber Neff (2024)	United States	Mixed Methods	ICU/palliative/oncology	Both	Anyone
Fauri and Grimes (1994)	United States	Descriptive	Psychiatric	Both	Anyone
Garland (2011)	New Zealand	Descriptive	ICU/palliative/oncology	Both	Anyone
Gibson et al. (2011)	United States	Qualitative	Peri/neonatal	Both	Parents
Goldberg et al. (2021)	United States	Qualitative	Paediatric	Both	Anyone
Grant et al. (2021)	Australia	Mixed Methods	Emergency/acute	Both	Anyone
Gundry et al. (2023)	Australia	Mixed Methods	ICU/palliative/oncology	Expected	Parents
			Paediatric		
Harding and Southern (1991)	United States	Descriptive	ICU/palliative/oncology	Expected	Anyone
Harmon et al. (1984)	United States	Mixed Methods	Peri/neonatal	Expected	Parents
Heiney et al. (1993)	United States	Qualitative	Paediatric	Both	Anyone
Holland and Rogich (1980)	United States	Descriptive	Emergency/acute	Sudden	Anyone
Hottensen (2013)	United States	Mixed Methods	Hospital-wide	Expected	Anyone
Jansen (2003)	United States	Descriptive	Peri/neonatal	Expected	Parents
Janson (1986)	United States	Qualitative	ICU/palliative/oncology	Expected	Anyone
Johnson et al. (1993)	United States	Descriptive	Paediatric	Both	Parents
Lake et al. (1983)	United States	Descriptive	Peri/neonatal	Sudden	Anyone
Lattanzi (1982)	United States	Descriptive	ICU/palliative/oncology	Expected	Anyone
LeBrocq et al. (2003)	Australia	Mixed Methods	Emergency/acute	Sudden	Anyone
Levick et al. (2017)	United States	Mixed Methods	Peri/neonatal	Expected	Anyone
McClelland (1993)	United States	Descriptive	ICU/palliative/oncology	Expected	Anyone
Modiba (2008)	South Africa	Descriptive	Peri/neonatal	Both	Parents
Morris and Block (2015)	United States	Quantitative	ICU/palliative/oncology	Expected	Anyone
Morris et al. (2017)	United States	Descriptive	Hospital-wide	Both	Anyone
			Paediatric		
Morris et al. (2023)	United States	Mixed Methods	Hospital-wide	Both	Anyone
Moseley et al. (1988)	United States	Descriptive	Paediatric	Both	Anyone
Nesbit et al. (1997)	United States	Mixed Methods	Paediatric	Both	Parents
Parris et al. (2007)	United Kingdom	Quantitative	Emergency/acute	Sudden	Anyone
Rappaport (1993)	Israel	Qualitative	Surgery	Both	Anyone
Reilly-Smorawski et al. (2002)	United States	Qualitative	Peri/neonatal	Both	Parents
Roose and Blanford (2011)	United States	Mixed Methods	Peri/neonatal	Both	Parents
Ross (2008)	United States	Qualitative	ICU/palliative/oncology	Both	Anyone
Ruden (1996)	United States	Mixed Methods	ICU/palliative/oncology	Expected	Parents
			Paediatric		
Ruetz (1989)	United States	Descriptive	Hospital-wide	Both	Anyone
Santiago et al. (2017)	Canada	Mixed Methods	ICU/palliative/oncology	Both	Anyone
Silloway et al. (2018)	United States	Descriptive	ICU/palliative/oncology	Expected	Anyone
Snaman et al. (2017)	United States	Mixed Methods	Paediatric	Expected	Parents
Snyder et al. (2002)	United States	Mixed Methods	Hospital-wide	Both	Anyone
Steen (2019)	United States	Quantitative	Peri/neonatal	Both	Parents
Tunnicliffe and Briggs (1997)	United Kingdom	Mixed Methods	ICU/palliative/oncology	Expected	Anyone
Vacha-Haase (2013)	United States	Descriptive	Paediatric	Expected	Anyone
			Emergency/acute		
Walsh et al. (2008)	Ireland	Mixed Methods	Hospital-wide	Both	Anyone
Weinfeld (1990)	United States	Quantitative	Peri/neonatal	Both	Parents
Weinhold (2007)	United States	Qualitative	Peri/neonatal	Both	Parents
Weiss et al. (1989)	United States	Descriptive	Peri/neonatal	Both	Parents
Williams et al. (2000)	Australia	Mixed Methods	Emergency/acute	Expected	Anyone
Williams et al. (2003)	United Kingdom	Mixed Methods	ICU/palliative/oncology	Expected	Anyone
Wintermeyer-Pingel et al. (2013)	United States	Quantitative	ICU/palliative/oncology	Both	Anyone
Witter et al. (1990)	United States	Quantitative	Hospital-wide	Both	Anyone
Yates et al. (1990)	United Kingdom	Qualitative	Emergency/acute	Sudden	Anyone

### Bereavement Models of Care

Thirteen studies provided information on the evidence used to develop the model of bereavement care. Evidence included literature review (Grant et al., 2021; Roose & Blanford, 2011; Ruden, 1996), review of existing local programs (Reilly-Smorawski et al., 2002), interviews with staff (Fauri & Grimes, 1994; Grant et al., 2021; Lattanzi, 1982; Morris et al., 2017; Moseley et al., 1988) and bereaved individuals (Berrett-Abebe et al., 2017; Edi-Osagie & Evans, 2005; Fauri & Grimes, 1994; Grant et al., 2021; Lattanzi, 1982; Morris et al., 2017), clinical experience (Gibson et al., 2011; Ross, 2008; Ruetz, 1989), and available research data on support services (Berrett-Abebe et al., 2017; Roose & Blanford, 2011). Table 3 summarizes the components within each model of care.

**Table 3.** Components of Bereavement Models of Care.

Study	Bereavement packs	Mementos/ memory making	Condolence card	Memorial service	Follow-up contact	Psycho-education	Peer support /support groups	Referrals	Community outreach	Bereavement counselling	Risk assessment	Staff training	Staff support	Translation/ interpretation/ cultural death norms	Evaluation
Adamowski et al. (1993)	Y	N	N	N	Y	N	N	N	N	N	N	Y	N	N	Y
Aho, Astedt-Kurki, Tarkka, & Kaunonen, (2011)	Y	N	N	N	Y	N	Y	N	N	N	N	Y	N	N	N
Aho, Astedt-Kurki, Tarkka, & Kaunonen, (2011)	Y	N	N	N	Y	N	N	N	N	N	N	Y	N	N	N
Berrett-Abebe et al. (2017)	Y	Y	N	N	Y	Y	N	Y	N	N	N	N	N	N	Y
Blackburn and Dwyer (2017)	N	N	N	N	N	N	N	N	N	N	Y	N	N	Y	N
Bucaro et al. (2005)	Y	Y	Y	Y	N	N	N	N	N	N	N	Y	N	Y	Y
Buchanan et al. (1996)	N	N	Y	Y	Y	N	N	Y	N	N	N	Y	Y	N	N
Burke and Gerraughty (1994)	Y	N	Y	Y	N	N	N	N	N	N	N	Y	N	Y	N
Chaplin (2003)	Y	N	Y	N	Y	N	N	N	N	N	N	Y	Y	N	N
Cole et al., 2020	Y	Y	Y	Y	Y	N	N	N	N	N	N	N	N	Y	Y
Coolican and Pearce (1995)	Y	N	Y	Y	Y	N	N	Y	N	N	Y	N	N	N	Y
Cooper et al. (2020)	Y	N	Y	N	Y	N	N	N	N	N	N	N	N	N	N
deJong-Berg and deVlaming (2005)	N	Y	Y	Y	Y	N	Y	N	N	N	N	Y	Y	Y	N
Edi-Osagie and Evans (2005)	Y	Y	N	Y	Y	Y	N	Y	N	Y	N	Y	Y	Y	Y
Edwards (2020)	Y	N	N	N	N	N	N	Y	N	N	N	N	N	N	N
Farrow and Felber Neff (2024)	N	Y	N	N	N	N	N	N	N	N	N	Y	Y	Y	N
Fauri and Grimes (1994)	Y	N	Y	N	Y	N	N	N	N	N	N	Y	N	N	N
Garland (2011)	Y	N	Y	N	Y	N	N	N	N	N	N	Y	Y	N	N
Gibson et al. (2011)	Y	Y	Y	Y	Y	Y	N	N	N	N	N	Y	Y	N	N
Goldberg et al. (2021)	Y	N	N	Y	N	N	Y	N	N	N	N	N	N	Y	Y
Grant et al., 2021	Y	N	Y	Y	Y	N	N	Y	N	N	Y	Y	N	Y	N
Gundry et al. (2023)	Y	N	Y	Y	Y	Y	Y	Y	Y	Y	N	N	Y	N	Y
Harding and Southern (1991)	N	N	N	N	N	Y	Y	N	Y	Y	N	Y	N	N	N
Harmon et al. (1984)	N	Y	N	N	Y	N	N	N	N	N	N	Y	N	N	Y
Heiney et al. (1993)	Y	N	Y	N	Y	Y	N	N	Y	N	Y	Y	Y	N	Y
Holland and Rogich (1980)	N	N	N	N	Y	N	N	N	N	Y	Y	N	N	N	N
Hottensen (2013)	Y	N	Y	Y	Y	N	Y	N	N	N	Y	Y	N	N	Y
Jansen (2003)	Y	Y	Y	Y	Y	N	N	N	N	Y	N	Y	N	Y	Y
Janson (1986)	Y	N	N	N	Y	Y	N	Y	Y	N	Y	Y	Y	N	N
Johnson et al. (1993)	Y	Y	Y	N	Y	N	N	N	N	N	N	N	N	N	N
Lake et al. (1983)	N	Y	N	N	Y	N	N	Y	N	N	Y	Y	N	N	N
Lattanzi (1982)	Y	N	Y	N	Y	Y	Y	Y	N	N	Y	Y	N	N	N
LeBrocq et al. (2003)	Y	N	Y	N	N	N	N	N	N	N	N	Y	N	Y	Y
Levick et al. (2017)	N	Y	Y	N	Y	N	Y	N	N	N	N	Y	N	Y	Y
McClelland (1993)	Y	N	Y	N	N	N	N	N	N	N	N	Y	N	N	Y
Modiba (2008)	N	Y	N	N	Y	N	N	N	N	Y	N	N	N	N	Y
Morris & Block, 2015	Y	N	Y	Y	Y	Y	Y	N	N	N	Y	Y	Y	N	Y
Morris et al. (2017)	Y	N	Y	Y	Y	Y	Y	Y	N	N	N	Y	Y	N	Y
Morris et al. (2023)	Y	N	Y	N	Y	Y	N	N	N	N	N	N	N	N	Y
Moseley et al. (1988)	Y	N	Y	N	Y	N	N	N	N	N	N	N	Y	N	N
Nesbit et al. (1997)	Y	Y	N	Y	Y	N	N	Y	N	N	Y	N	N	N	Y
Parris et al. (2007)	Y	N	N	N	Y	N	N	Y	N	N	Y	N	N	N	Y
Rappaport (1993)	N	N	N	N	N	N	N	Y	N	Y	Y	N	N	Y	Y
Reilly-Smorawski et al. (2002)	N	N	Y	Y	Y	N	Y	N	N	N	N	N	N	N	Y
Roose and Blanford (2011)	N	Y	N	N	N	N	Y	N	N	N	N	N	N	Y	N
Ross (2008)	Y	N	Y	N	Y	N	N	Y	N	N	N	N	N	N	Y
Ruden (1996)	Y	N	Y	N	Y	Y	Y	N	N	N	N	N	N	N	Y
Ruetz (1989)	Y	N	Y	Y	N	N	Y	N	N	N	N	N	N	N	N
Santiago et al. (2017)	Y	N	Y	Y	Y	N	N	N	N	N	N	N	N	N	Y
Silloway et al. (2018)	Y	N	Y	Y	Y	Y	N	N	N	N	N	N	Y	N	N
Snaman et al. (2017)	Y	Y	Y	Y	N	N	Y	N	N	N	Y	Y	N	N	Y
Snyder et al. (2002)	Y	Y	Y	Y	N	N	Y	N	N	N	N	N	N	N	Y
Steen (2019)	N	Y	Y	Y	Y	N	N	N	N	N	N	Y	Y	N	Y
Tunnicliffe and Briggs (1997)	N	N	Y	N	Y	N	N	N	N	N	N	Y	N	N	Y
Vacha-Haase (2013)	N	N	Y	N	N	N	Y	N	N	N	N	N	N	N	Y
Walsh et al. (2008)	Y	N	N	Y	N	N	Y	Y	Y	Y	N	Y	N	N	Y
Weinfeld (1990)	Y	Y	N	N	Y	N	Y	Y	N	N	N	Y	Y	N	N
Weinhold (2007)	N	N	N	N	Y	N	Y	N	N	N	N	N	N	N	N
Weiss et al. (1989)	Y	N	N	N	N	Y	Y	N	Y	Y	Y	Y	N	N	N
Williams et al. (2000)	Y	N	Y	N	Y	N	N	N	N	N	N	Y	N	N	N
Williams et al. (2000)	Y	N	Y	N	Y	N	Y	N	N	N	N	N	N	Y	Y
Wintermeyer-Pingel et al. (2013)	Y	N	Y	N	N	N	Y	N	Y	N	N	Y	Y	N	N
Witter et al. (1990)	Y	N	Y	N	Y	N	N	N	N	N	N	Y	Y	Y	N
Yates et al. (1990)	N	N	N	N	Y	N	N	N	N	Y	Y	Y	Y	Y	Y

#### Services Provided at Time of Death

The most common component of the models of care was provision of bereavement packs that were handed out at the time of death. These included information on grief and bereavement, practical supports such as funeral arrangements or organ donation, and available support and how to access it. Another common component was memory making activities for the bereaved. Mementos included hand or foot molds, photographs, personal items, toys, lock of hair, or quilts. These activities were mostly delivered in peri/neonatal or pediatric settings.

#### Follow up Care Provided

Follow-up care consisted of condolence card(s) given shortly after death, but also on key milestone events (e.g., birthday of the deceased, anniversary of death). Memorial services, either unit specific or hospital wide, were another commonly included component of bereavement models of care. Follow-up contact, via phone calls, visits, and/or family meetings were either on a single occasion following the death or multiple occasions spanning the course of a year. Less common forms of support included psycho-education, which included educational programs, educational resources, education offered in routine follow-up, as well as peer support, referrals to specialized services and community outreach, and bereavement counselling.

#### Bereavement Assessment Tools

Only 14 studies incorporated a formal assessment (tool) for complex grief, such as the Bereavement Common Assessment Framework (Blackburn & Dwyer, 2017), the Colorado Hospice Bereavement Assessment Tool (Hottensen, 2013), Parkes’ Determinants of Grief risk factor scale (Janson, 1986; Lattanzi, 1982), the Grief Experience Inventory (Janson, 1986) or self-developed assessment tools (Coolican & Pearce, 1995; Grant et al., 2021; Heiney et al., 1993; Holland & Rogich, 1980; Lake et al., 1983; Nesbit et al., 1997; Parris, Schlosenberg, Stenley, Maurice, & Clarke, 2007; Snaman et al., 2017; Weiss et al., 1989; Yates et al., 1990). The other models of care either included informal assessments integrated into the follow-up calls (Fauri & Grimes, 1994; Garland, 2011; Harmon et al., 1984; Jansen, 2003; Modiba, 2008; Morris & Block, 2015; Moseley et al., 1988; Ross, 2008; Santiago et al., 2017; Walsh et al., 2008; Weinfeld, 1990; Weinhold, 2007; Wintermeyer-Pingel et al., 2013; Witter et al., 1990; Yates et al., 1990) or no risk assessment.

#### Staff Education and Support

Most bereavement models of care included an element of staff education which ranged from a single training session to multi-week education programs. Two studies also included providing education to the community to improve knowledge about grief (Weinfeld, 1990; Weiss et al., 1989). Fewer studies included support for staff who deliver bereavement care as a component of their model of bereavement care.

#### Culturally Inclusive Practices

Seventeen studies mentioned multi-cultural, multi-faith or multi-lingual considerations in the description of their bereavement models of care (Blackburn & Dwyer, 2017; Bucaro et al., 2005; Burke & Gerraughty, 1994; Cole et al., 2020; deJong-Berg & deVlaming, 2005; Edi-Osagie & Evans, 2005; Farrow & Felber Neff, 2024; Goldberg et al., 2021; Grant et al., 2021; Jansen, 2003; LeBrocq et al., 2003; Levick et al., 2017; Rappaport, 1993; Roose & Blanford, 2011; Williams et al., 2003; Witter et al., 1990; Yates et al., 1990). However, these considerations were mostly brief and nonspecific, such as bereavement cards that were neutral and could be used cross-culturally.

#### Evaluation and Continual Improvement

Finally, data collection, evaluation and continual improvement were also included in descriptions of some models of bereavement care. This included feedback from bereaved and/or staff, ranging from spontaneous feedback to a one-off survey, to ongoing audits or evaluations. Few studies explicitly stated that revisions to the program were made based on feedback (Coolican & Pearce, 1995; Goldberg et al., 2021; Gundry et al., 2023; Witter et al., 1990). Three studies reported the evaluation of their bereavement programs (Berrett-Abebe et al., 2017; Fauri & Grimes, 1994; Morris et al., 2023).

### Bereavement Models of Care Implementation

#### Barriers to Provision of Bereavement Care

Potential challenges to bereaved individuals accessing bereavement care included limited access due to geographical distance (Berrett-Abebe et al., 2017; Gundry et al., 2023; Morris & Block, 2015; Morris et al., 2017; Reilly-Smorawski et al., 2002), other caring responsibilities (Reilly-Smorawski et al., 2002), timing of bereavement care too soon after death (Ruetz, 1989), and/or bereaved individuals having to return to the place of their loved ones death to receive care (Berrett-Abebe et al., 2017; Edi-Osagie & Evans, 2005; Gundry et al., 2023; Morris et al., 2017; Ruetz, 1989). At a service level, a lack of institutional and financial support (Berrett-Abebe et al., 2017; Farrow & Felber Neff, 2024; Gibson et al., 2011; Morris et al., 2023; Silloway et al., 2018; Snaman et al., 2017); difficulty identifying bereaved individuals, and collecting and maintaining contact information (Morris & Block, 2015; Morris et al., 2017; Snaman et al., 2017); large caseloads of bereaved individuals (Morris & Block, 2015); difficulty communicating and coordinating bereavement services offered across departments (Snaman et al., 2017); identifying an acceptable risk assessment tool (Morris & Block, 2015; Snaman et al., 2017); staff discomfort with delivering bereavement care (Farrow & Felber Neff, 2024; Holland & Rogich, 1980; Ruden, 1996; Snaman et al., 2017; Tunnicliffe & Briggs, 1997); lack of time and/or dedicated staff (Berrett-Abebe et al., 2017; Farrow & Felber Neff, 2024; Gibson et al., 2011; Vacha-Haase, 2013); and concerns related to lack of staff training and impact of staff wellbeing, including risk of burnout and turnover (Ruden, 1996; Snaman et al., 2017) were also barriers.

#### Organizational Commitment to Implementation

Conversely, adequate institutional (Gibson et al., 2011; Moseley et al., 1988; Steen, 2019; Witter et al., 1990) or community (Gibson et al., 2011; Weinfeld, 1990) funding allowed for a dedicated bereavement budget (Edi-Osagie & Evans, 2005; Moseley et al., 1988; Witter et al., 1990) to fund components of the bereavement model of care (Steen, 2019) as well fund dedicated staff to coordinate or deliver the model of care (Edi-Osagie & Evans, 2005; Steen, 2019) were reported as facilitators to implementation. Infrastructure such as dedicated spaces for death and dying conversations or grief to occur (Farrow & Felber Neff, 2024; Harmon et al., 1984; R. Williams et al., 2003; Witter et al., 1990; Yates et al., 1990); and developing systems to track provision of bereavement services provided and preferences of the bereaved (Cole et al., 2020; Cooper et al., 2020; Garland, 2011; Modiba, 2008; Morris & Block, 2015; Moseley et al., 1988; Snaman et al., 2017; Tunnicliffe & Briggs, 1997); communication between teams (Modiba, 2008; Yates et al., 1990), with primary care physicians or general practitioners (Yates et al., 1990), and with community services (Yates et al., 1990) all contributed to service delivery (Table 4).

**Table 4.** Strategies to Facilitate Implementation of Bereavement Models of Care.

Study	Funding & infrastructure	Policies, protocols & procedures	Whole of service approach	Co-design /delivered	Tailoring	Referral pathways & links to resources	Dedicated/ adequate staffing, roles & responsibilities	Engagement & awareness campaign	Risk assessment	Training	Staff/ Volunteer support
Adamowski et al. (1993)	N	Y	N	N	N	Y	N	Y	N	Y	N
Aho, Astedt-Kurki, Tarkka, & Kaunonen, (2011)	N	Y	N	N	N	N	N	Y	N	Y	N
Aho, Tarkka, Åstedt-Kurki, & Kaunonen (2011)	N	N	N	N	N	N	N	N	N	Y	N
Berrett-Abebe et al. (2017)	N	Y	N	Y	N	N	Y	N	N	N	N
Bucaro et al. (2005)	N	Y	N	N	N	N	N	Y	N	Y	N
Buchanan et al. (1996)	N	Y	N	N	N	N	N	N	N	N	N
Burke and Gerraughty (1994)	N	N	N	N	N	N	N	N	N	Y	N
Chaplin (2003)	N	Y	Y	N	Y	Y	Y	N	N	Y	N
Cole et al., 2020	Y	Y	N	N	N	N	Y	N	N	N	N
Coolican and Pearce (1995)	N	Y	N	N	N	N	Y	N	N	N	N
Cooper et al. (2020)	Y	Y	N	N	N	N	Y	N	N	N	N
deJong-Berg and deVlaming (2005)	N	Y	Y	N	N	N	Y	N	N	Y	Y
Edi-Osagie and Evans (2005)	N	N	Y	N	y	Y	Y	N	N	Y	Y
Edwards (2020)	N	Y	N	N	N	N	Y	N	N	N	N
Farrow and Felber Neff (2024)	Y	N	N	N	N	N	Y	N	N	Y	
Fauri and Grimes (1994)	N	N	N	N	N	N	N	N	N	Y	N
Garland (2011)	Y	N	N	N	N	N	N	N	N	N	N
Gibson et al. (2011)	Y	Y	Y	N	N	N	Y	N	N	Y	Y
Goldberg et al. (2021)	N	Y	N	N	N	N	N	N	N	N	N
Grant et al., 2021	N	N	N	N	N	N	Y	N	N	Y	N
Gundry et al. (2023)	N	N	N	N	N	Y	Y	N	N	Y	Y
Harmon et al. (1984)	Y	N	N	N	N	N	N	N	N	N	N
Heiney et al. (1993)	N	Y	N	N	N	N	N	Y	N	Y	N
Hottensen (2013)	N	N	N	N	N	N	Y	N	N	N	N
Holland and Rogich (1980)	N	Y	N	N	N	Y	Y	N	N	N	N
Jansen (2003)	N	Y	N	N	N	N	N	N	N	N	N
Johnson et al. (1993)	N	Y	N	N	N	N	Y	Y	N	N	N
Lake et al. (1983)	N	Y	N	N	N	N	N	N	N	N	N
Lattanzi (1982)	N	N	N	N	N	N	Y	N	N	Y	N
LeBrocq et al. (2003)	N	N	N	N	N	N	N	Y	N	N	N
McClelland (1993)	N	Y	Y	N	Y	N	Y	N	N	N	N
Modiba (2008)	Y	Y	Y	N	N	N	N	N	N	Y	Y
Morris et al. (2023)	Y	Y	N	N	Y	Y	Y	N	Y	Y	Y
Morris et al. (2017)	N	Y	N	N	Y	Y	Y	Y	N	Y	Y
Morris et al. (2023)	N	N	N	N	N	N	N	N	N	N	N
Moseley et al. (1988)	Y	Y	N	N	Y	N	Y	Y	N	Y	N
Nesbit et al. (1997)	N	N	N	N	N	N	Y	N	N	Y	Y
Parris et al. (2007)	N	N	N	N	N	Y	N	N	Y	N	N
Rappaport (1993)	N	N	N	N	Y	N	Y	N	Y	N	N
Reilly-Smorawski et al. (2002)	N	Y	N	N	N	N	N	N	N	N	N
Roose and Blanford (2011)	N	N	N	N	Y	N	Y	N	N	Y	N
Ross (2008)	N	N	N	N	Y	N	N	N	N	N	N
Ruden (1996)	N	Y	N	Y	N	N	Y	Y	N	Y	N
Ruetz (1989)	N	N	N	N	N	N	Y	N	N	N	N
Silloway et al. (2018)	N	Y	Y	N	N	N	Y	N	N	N	N
Snaman et al. (2017)	Y	Y	Y	Y	N	Y	Y	N	N	Y	Y
Snyder et al. (2002)	N	N	Y	N	N	N	N	N	N	N	N
Steen (2019)	Y	Y	N	N	N	N	Y	N	N	Y	Y
Tunnicliffe and Briggs (1997)	Y	Y	Y	N	N	N	Y	Y	N	Y	Y
Vacha-Haase (2013)	N	Y	N	N	N	N	N	N	N	N	N
Weinfeld (1990)	Y	Y	Y	Y	N	Y	N	N	N	Y	Y
Weinhold (2007)	N	Y	N	N	N	N	Y	N	N	N	N
Weiss et al. (1989)	N	Y	Y	N	N	N	N	N	Y	Y	Y
Williams et al. (2000)	N	N	N	N	N	N	N	N	N	Y	N
Williams et al. (2003)	Y	N	Y	N	N	N	N	N	N	Y	Y
Wintermeyer-Pingel et al. (2013)	N	N	Y	N	N	N	N	N	N	Y	Y
Witter et al. (1990)	Y	Y	Y	N	N	N	N	N	N	Y	Y
Yates et al. (1990)	N	N	N	N	N	Y	Y	N	Y	Y	Y

At an organizational level, establishing policies that provided standardized protocols and procedures for staff to follow also facilitated the implementation of bereavement models of care (Table 4) including timelines for care delivery (Tunnicliffe & Briggs, 1997; Vacha-Haase, 2013; Weiss et al., 1989) and guidelines and templates to assist staff (Gibson et al., 2011; Holland & Rogich, 1980; Reilly-Smorawski et al., 2002; Ruden, 1996; Tunnicliffe & Briggs, 1997; Weinfeld, 1990; Witter et al., 1990) in delivering bereavement care. Four studies also described changing existing organizational policies (Modiba, 2008; Moseley et al., 1988; Steen, 2019; Witter et al., 1990) to allow flexibility in visiting hours (Modiba, 2008), increased privacy (Modiba, 2008), extending the time that the deceased can remain on ward to allow families to view the body (Moseley et al., 1988; Witter et al., 1990), or moving bereaved mothers to other wards to avoid reminders of their loss (crying babies, teddy bears) (Modiba, 2008).

Studies reported a whole of service approach to the development and delivery of bereavement care (Table 4), through regular meetings to discuss bereavement care, staff support (Modiba, 2008), and to ensure communication between wards/departments, to address any issues, and facilitate continuity of care (Chaplin, 2003; Modiba, 2008; Tunnicliffe & Briggs, 1997; Witter et al., 1990). Involving bereaved individuals in the model of care development, co-design of staff training, and as peer support, were also perceived to facilitate implementation of bereavement models of care (Table 4).

Aligning or tailoring the model of care to the needs of specific patient and bereaved populations (McClelland, 1993; Morris & Block, 2015; Morris et al., 2017), was reported in three studies as a strategy to aid implementation. Six studies also recognized the importance of tailoring bereavement care for culturally and linguistically diverse populations (Chaplin, 2003; Edi-Osagie & Evans, 2005; Moseley et al., 1988; Rappaport, 1993; Roose & Blanford, 2011; Ross, 2008), including resources/services in translation, use of interpreters, and recognizing cultural death norms.

Having established referral pathways to other services within the health system (Chaplin, 2003; Edi-Osagie & Evans, 2005; Gundry et al., 2023; Holland & Rogich, 1980; Parris et al., 2007; Weinfeld, 1990) or community resources (Adamowski et al., 1993; Morris & Block, 2015; Yates et al., 1990), as well as having resources (Morris et al., 2017; Snaman et al., 2017; Weinfeld, 1990) and information about bereavement services (Morris & Block, 2015; Morris et al., 2017; Snaman et al., 2017) publicly available, was considered a facilitator to bereaved individuals accessing information and support. Five studies also mentioned screening for risk (where possible) of PGD as important (Table 4).

#### Staff Responsibilities and Engagement

Adequate staffing to deliver bereavement model of care was considered important (Chaplin, 2003; Edwards, 2020) during the development of the models of bereavement care. Studies reported the need for staff or volunteer roles dedicated to delivering or coordinating bereavement care (Tabe 3), including bereavement care officers/coordinators (Chaplin, 2003; Coolican & Pearce, 1995; Grant et al., 2021; Johnson et al., 1993; McClelland, 1993; Morris et al., 2017; Roose & Blanford, 2011; Weinhold, 2007), bereavement care managers/directors (Chaplin, 2003; Morris et al., 2017), or by incorporating bereavement care responsibilities into existing roles. Where bereavement care was incorporated into existing roles, it was considered important to ensure that bereavement responsibilities (McClelland, 1993; Rappaport, 1993; Ruetz, 1989; Tunnicliffe & Briggs, 1997; Weinhold, 2007) were formalized and clearly defined. Further, one study suggested attributes of bereavement staff, such as communication and interpersonal skills, comfortable with grief and loss, and diversity would facilitate bereavement care delivery (Lattanzi, 1982).

Four studies discussed the importance of staff seeing value in and supporting bereavement care (Heiney et al., 1993; Johnson et al., 1993; Moseley et al., 1988; Ruden, 1996) to facilitate acceptance and ownership of the model of care. Awareness campaigns including in-service sessions were commonly used to introduce staff and volunteers to the model of care and its implementation at their service (Adamowski et al., 1993; Aho, Astedt-Kurki, Tarkka, & Kaunonen, 2011; Heiney et al., 1993; Johnson et al., 1993; Tunnicliffe & Briggs, 1997), Posters (Bucaro et al., 2005; LeBrocq et al., 2003), group or one-to-one presentations (Heiney et al., 1993; LeBrocq et al., 2003), letters (Heiney et al., 1993), newsletters (Heiney et al., 1993), department announcements (Heiney et al., 1993), information brochures (LeBrocq et al., 2003), and pamphlets in staff orientation packs and on staff intranet (LeBrocq et al., 2003) were also used to promote the model of care to staff. Additionally, identification of a champion for the model of care (Morris et al., 2017) or having support from the researchers throughout the implementation (Aho, Astedt-Kurki, Tarkka, & Kaunonen, 2011) were factors that facilitated implementation.

Education and training were well documented strategies to facilitate implementation (Table 4). Education and training aimed to improve knowledge of death, bereavement, and grief (Adamowski et al., 1993; Aho, Astedt-Kurki, Tarkka, & Kaunonen, 2011; Bucaro et al., 2005; Burke & Gerraughty, 1994; Chaplin, 2003; Fauri & Grimes, 1994; Heiney et al., 1993; Modiba, 2008; Morris & Block, 2015; Morris et al., 2017; Nesbit et al., 1997; Roose & Blanford, 2011; Ruden, 1996; Snaman et al., 2017; Weinfeld, 1990), the model of care (Burke & Gerraughty, 1994; Modiba, 2008; Morris & Block, 2015; Tunnicliffe & Briggs, 1997), how to deliver bereavement care (Aho, Astedt-Kurki, Tarkka, & Kaunonen, 2011; Aho, Tarkka, Åstedt-Kurki, & Kaunonen, 2011; Edi-Osagie & Evans, 2005; Gibson et al., 2011; Modiba, 2008; Morris et al., 2017; Snaman et al., 2017), cultural and religious requirements (Chaplin, 2003; Snaman et al., 2017; Weinfeld, 1990), communication skills (Grant et al., 2021; Weinfeld, 1990; Witter et al., 1990), and self-care strategies (Burke & Gerraughty, 1994; Morris & Block, 2015; Morris et al., 2017; Snaman et al., 2017; Weinfeld, 1990). Three studies highlighted education/training (Morris & Block, 2015; Morris et al., 2017; Wintermeyer-Pingel et al., 2013) was provided regularly, and another integrated education into staff induction packs to capture new staff (Gibson et al., 2011; Tunnicliffe & Briggs, 1997).

Routine support for staff/volunteers providing bereavement services was another well documented strategy to facilitate implementing bereavement models of care (Table 4). Support strategies included risk assessments (Snaman et al., 2017), bereavement rounds (Morris & Block, 2015; Wintermeyer-Pingel et al., 2013), de-briefing (deJong-Berg & deVlaming, 2005; Gibson et al., 2011; Morris & Block, 2015; Morris et al., 2017; Nesbit et al., 1997; Snaman et al., 2017; Williams et al., 2003), reflective journal keeping (Modiba, 2008), support groups (Modiba, 2008; Tunnicliffe & Briggs, 1997; Yates et al., 1990), counselling (Gibson et al., 2011; Yates et al., 1990) and supervision (Weinfeld, 1990; Weiss et al., 1989).

Most of the literature focussed on describing bereavement models of care, the potential challenges, and strategies to facilitate implementation. Fourteen studies further reported implementation data collection processes (Aho, Astedt-Kurki, Tarkka, & Kaunonen, 2011; Aho, Tarkka, Åstedt-Kurki, & Kaunonen, 2011; LeBrocq et al., 2003; McClelland, 1993; Santiago et al., 2017; Tunnicliffe & Briggs, 1997) and/or outcomes (Aho, Tarkka, Åstedt-Kurki, & Kaunonen, 2011; Berrett-Abebe et al., 2017; Bucaro et al., 2005; Farrow & Felber Neff, 2024; Gundry et al., 2023; Johnson et al., 1993; LeBrocq et al., 2003; McClelland, 1993; Morris et al., 2023; Ruden, 1996; Santiago et al., 2017; Tunnicliffe & Briggs, 1997; A. Williams et al., 2000).

##### Implementation Data collection

Implementation data was collected through questionnaires with staff/volunteers (Aho, Astedt-Kurki, Tarkka, & Kaunonen, 2011; LeBrocq et al., 2003; Santiago et al., 2017) and families (Santiago et al., 2017), interviews with staff (Aho, Astedt-Kurki, Tarkka, & Kaunonen, 2011), an audit (Tunnicliffe & Briggs, 1997), and anecdotal letters, visits, and comments from families (LeBrocq et al., 2003; McClelland, 1993). Data collection assessed training sessions (Aho, Astedt-Kurki, Tarkka, & Kaunonen, 2011), awareness and perceptions of the model of care

(LeBrocq et al., 2003), experiences of implementation (Aho, Astedt-Kurki, Tarkka, & Kaunonen, 2011; Aho, Tarkka, Åstedt-Kurki, & Kaunonen, 2011; LeBrocq et al., 2003), feasibility (Santiago et al., 2017), acceptability (Santiago et al., 2017), and suggestions for future improvements (Aho, Tarkka, Åstedt-Kurki, & Kaunonen, 2011; LeBrocq et al., 2003). Some of these studies while outlining their implementation outcome data collection, did not report implementation outcomes in the same study (Aho, Astedt-Kurki, Tarkka, & Kaunonen, 2011).

#### Implementation Outcomes

##### Acceptability

Studies reported staff receiving the models of care with enthusiasm and appreciation (Farrow & Felber Neff, 2024; Johnson et al., 1993; LeBrocq et al., 2003; Tunnicliffe & Briggs, 1997). Specifically, staff expressed positive responses to the in-service (Bucaro et al., 2005), poster (Bucaro et al., 2005), and formal training (Aho, Tarkka, Åstedt-Kurki, & Kaunonen, 2011; LeBrocq et al., 2003; A. Williams et al., 2000). One study further reported that staff found the content and distribution of bereavement packs acceptable, felt that bereaved individuals were receptive to follow-up and reported positive experiences in delivering care (Aho, Tarkka, Åstedt-Kurki, & Kaunonen, 2011). Another study reported that staff indicated greater ability and confidence in interacting with bereaved (Johnson et al., 1993).

Studies reporting informal feedback from relatives reported that the models of bereavement care were positively received by bereaved individuals (Berrett-Abebe et al., 2017; Gundry et al., 2023; LeBrocq et al., 2003; McClelland, 1993; Morris et al., 2023; Ruden, 1996; Tunnicliffe & Briggs, 1997; A. Williams et al., 2000). However, Santiago et al. (2017) reported that only 44% of bereaved survey respondents rated their care as acceptable.

##### Feasibility

Feasibility outcomes were reported in two studies. Santiago et al. (2017) reported that their feasibility target was met for three components of their model of care, with greater than 75% of eligible families receiving sympathy cards, bereavement brochures, and invitations to the memorial service, but not for the follow up phone call component of the model of care. Aho, Tarkka, Åstedt-Kurki, & Kaunonen (2011) reported that staff felt that the timing of follow-up care was realistic, information transfer to peer supporters was viable. Staff also reported that the versatility of follow-up contact (phone, meeting, email, letter, text message) was useful, but that phone and family meetings were the most used.

##### Fidelity

Tunnicliffe and Briggs (1997) reported an increase in bereavement follow-up compared with previous years and that in 95% of instances nurses adhered to the standards.

## Discussion

This systematic review reports an overview of peer-reviewed published models of bereavement care and implementation factors. The findings of this systematic review build on previous reviews which have focussed on bereavement standards and guidelines (Hudson et al., 2018), or bereavement support provided in specific contexts (Efstathiou et al., 2019; Erikson & McAdam, 2020). Specifically, our review summarises models of bereavement care, potential facilitators and barriers to implementation and implementation outcomes.

This review described models of bereavement care that commenced at and post death. The level of care provided was typically universal care (e.g., condolence cards, follow-up calls/visits, memorial services), and are commonly reported services offered to bereaved individuals (Boven et al., 2022). However, few studies discussed the evidence used to develop their models of bereavement care.

Few studies described the process for identifying bereaved individuals at risk of prolonged grief and the care provided or referral pathways for these individuals. Aoun et al. (2012) proposed a public health approach to bereavement in palliative care which is underpinned by meeting the needs of bereaved individuals while remaining cost-effective. This proposed approach advocates for stepped care, with universal care (e.g., information about bereavement and relevant supports) to all bereaved, non-specialist support for bereaved individuals at risk of developing complex needs, and specialist intervention for bereaved individuals with complex needs. This stepped care approach highlights a need for further research into the best approach to screening bereaved individuals if risk stratification is to be used to inform bereavement care (Hay et al., 2021).

The majority of bereavement models of care included in this review were developed and/or implemented in palliative care, highlighting a need for further research in other health settings to better understand the unique bereavement needs across types of deaths (e.g., expected vs. sudden) and bereaved populations and facilitate tailoring of the model of care to address the needs of all bereaved individuals in the health service (Aoun et al., 2012).

Another gap identified through this review was the limited evidence on appropriate bereavement care for culturally and linguistically diverse communities. This supports a recent review of bereavement care for ethnic minorities in the UK (Mayland et al., 2021) which found a lack of understanding regarding grief and bereavement in different languages, cultures and religion. Further, this review found that there was no research reporting satisfaction with, or outcomes of accessing bereavement support.

While most studies discussed the potential challenges and facilitators considered during the development of the model of care, few studies included implementation data collection and reported implementation outcomes. Those reporting implementation outcomes focussed on acceptability, facilitators, and barriers, with little on feasibility and fidelity. Feasibility outcomes are important to understand the extent to which the proposed model of care can be integrated within the service, and fidelity is important to understand the degree to which the model of care was implemented as intended (Proctor et al., 2011). Future research should consider implementation frameworks during the development of bereavement models of care, incorporate implementation strategies into the model of care, and report implementation outcomes.

### Strengths and Limitations

A strength of this review is the inclusion of implementation factors. Consolidating the evidence base of factors that can influence implementation of bereavement care is useful to health care providers and organisations and can inform implementation strategies and activities. Our search strategy was designed to capture bereavement models of care developed for and/or implemented into health service settings. The findings of this review are not generalisable to other settings, where bereavement care is also delivered such as community-based settings. A further limitation of this research was the restriction of English language publications, which consequently limited the inclusion of bereavement models of care to those developed and implemented in high income Western countries such as the United States of America, Australia, and the United Kingdom.

## Conclusion

This review provides an overview of bereavement models of care, interventions commonly included in bereavement models of care, and barriers and facilitators in bereavement model of care development. Research across health settings and consideration of unique aspects of care for culturally and linguistically diverse populations, would further enhance understanding of bereavement care. As implementation and outcomes were rarely discussed, future research that embeds implementation theory and frameworks into model of care design and implantation are needed.
